# GADD45A is a protective modifier of neurogenic skeletal muscle atrophy

**DOI:** 10.1172/jci.insight.149381

**Published:** 2021-07-08

**Authors:** Jeffrey T. Ehmsen, Riki Kawaguchi, Damlanur Kaval, Anna E. Johnson, Daniel Nachun, Giovanni Coppola, Ahmet Höke

**Affiliations:** 1 Neuromuscular Division, Department of Neurology, Johns Hopkins School of Medicine, Baltimore, Maryland, USA.; 2 Department of Neurology and Department of Psychiatry and Biobehavioral Sciences, David Geffen School of Medicine, University of California, Los Angeles, Los Angeles, California, USA.

**Keywords:** Muscle Biology, Neuroscience, Neuromuscular disease, Skeletal muscle

## Abstract

Neurogenic muscle atrophy is the loss of skeletal muscle mass and function that occurs with nerve injury and in denervating diseases, such as amyotrophic lateral sclerosis. Aside from prompt restoration of innervation and exercise where feasible, there are currently no effective strategies for maintaining skeletal muscle mass in the setting of denervation. We conducted a longitudinal analysis of gene expression changes occurring in atrophying skeletal muscle and identified growth arrest and DNA damage-inducible A (*Gadd45a*) as a gene that shows one of the earliest and most sustained increases in expression in skeletal muscle after denervation. We evaluated the role of this induction using genetic mouse models and found that mice lacking GADD45A showed accelerated and exacerbated neurogenic muscle atrophy, as well as loss of fiber type identity. Our genetic analyses demonstrate that, rather than directly contributing to muscle atrophy as proposed in earlier studies, GADD45A induction likely represents a protective negative feedback response to denervation. Establishing the downstream effectors that mediate this protective effect and the pathways they participate in may yield new opportunities to modify the course of muscle atrophy.

## Introduction

Skeletal muscle atrophy is the decline of muscle mass and function resulting from degradation of contractile proteins and corresponding reduction in individual myofiber size. Muscle atrophy can result from many causes, including disuse, glucocorticoid use, cancer cachexia, or denervation.

The decrease in myofiber size and contractile function observed in muscle atrophy reflects the active degradation of contractile proteins such as myosin and actin, which occurs largely through induction of muscle-specific ubiquitin ligases and targeted proteasomal degradation (1, 2). Lysosomal proteolysis is also thought to contribute (3). Activation of members of the forkhead box O and NF-κB transcription factor families is thought to coordinate these processes, although the proximal events triggered by denervation that lead to these degradative events are not well characterized (4, 5). Muscle atrophy has substantial consequences, reflected in the relationship between functional capacity and quality of life in mild or moderate denervating disease, in the relationship between body mass index and survival in the severe denervation of amyotrophic lateral sclerosis (ALS), and in the impaired capacity for chronically denervated and severely atrophied muscle to be functionally reinnervated (6, 7).

Growth arrest and DNA damage-inducible A (GADD45A) was first identified in a Chinese hamster ovary–based screen for early response genes involved in the DNA damage response to ultraviolet (UV) irradiation (8). It is now known to be a member of a family of small, highly conserved, stress-inducible acidic nuclear proteins with ubiquitously low or absent expression that show cell type–specific induction in response to cellular stress. GADD45A and its related family members (GADD45B and GADD45C) do not appear to have enzymatic activity but exert their effects in response to cell stress by modulating the function of a wide variety of binding partners, with pleiotropic effects related to cellular senescence, apoptosis, cell cycle arrest, DNA repair, and epigenetic modification (9).

We performed a longitudinal analysis of gene expression changes in denervated, atrophying gastrocnemius muscle using a mouse model of tibial nerve transection and identified *Gadd45a* as the most significantly differentially expressed transcript at 1 day postdenervation, with significantly sustained induction through at least 90 days postdenervation. Using conventional and conditional mouse models of targeted *Gadd45a* gene deletion, we found that absence of *Gadd45a* induction in the setting of denervation was associated with significantly accelerated and exacerbated skeletal muscle atrophy compared with WT or heterozygous littermates. These observations indicate that *Gadd45a* induction after denervation mediates a protective effect during muscle atrophy and suggest that identification of effectors of this response may reveal new targets to modify the course of atrophic disease.

## Results

### Gadd45a induction is an acute and sustained response to denervation injury.

Several transcription factors and proteins involved in coordinating the degradative events of muscle atrophy have been defined, but how these events are triggered by denervation, how they interact and intersect, and how they change over time in relation to the phenotype of chronically atrophied muscle is not well understood. We used RNA-Seq to characterize changes in mRNA expression occurring in mouse gastrocnemius following tibial nerve transection. We performed a longitudinal analysis capturing transcriptional changes occurring acutely after denervation (1 day postdenervation), early changes during the window of most rapid atrophy (3, 7, and 14 days postdenervation), and changes associated with chronic denervation (30 and 90 days postdenervation) (10).

**Figure 1:**
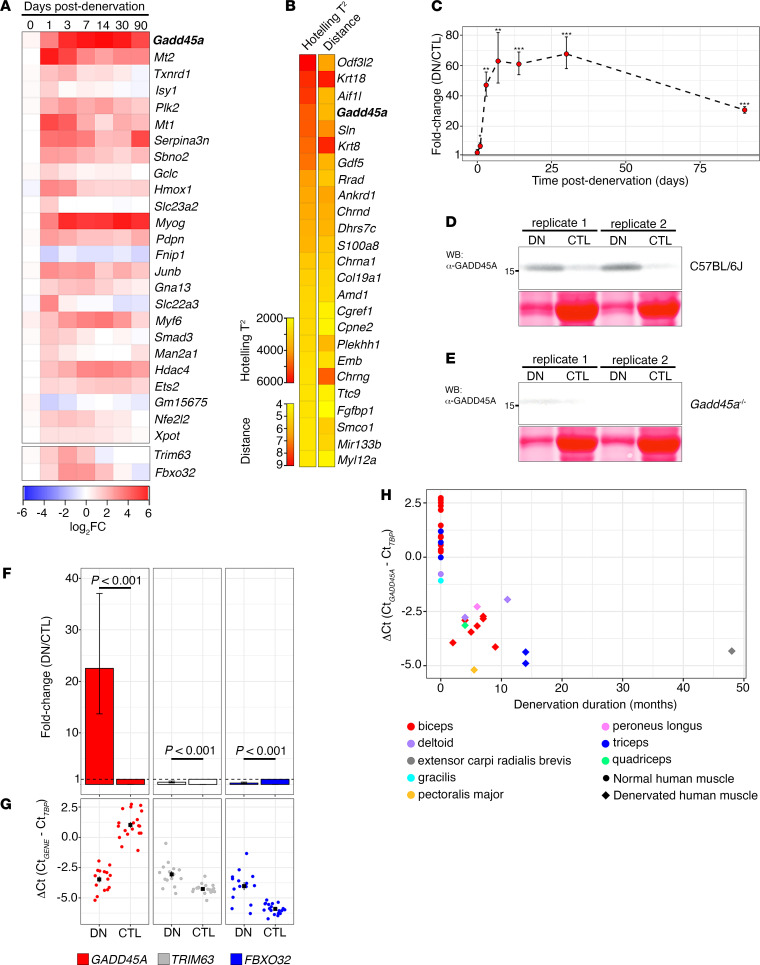
*Gadd45a* induction is an acute and sustained response to denervation injury. *Gadd45a* is the most significantly differentially expressed transcript in acutely denervated mouse gastrocnemius and shows sustained induction up to 3 months postdenervation (DN) (**A**–**D**; **P < 0.01, ***P < 0.001, paired *t* test with Bonferroni’s correction, *n* *=* 4 for each time point). GADD45A is similarly induced in a diverse range of denervated human skeletal muscle from individuals with acute denervation injury or acute flaccid myelitis and remains elevated up to 4 years after denervation (**F**–**H**, Welch’s *t* test). Error bars in **F** show mean ± SEM. All the normalized *GADD45A* threshold detection cycles (ΔCt) from denervated skeletal muscle are substantially lower than controls, indicating higher mean expression levels (**H**).

We observed that *Gadd45a* showed one of the earliest, largest in magnitude, and most sustained changes in expression following denervation, as determined using a limma-voom paired analysis of gene expression changes among denervated and contralateral intact gastrocnemii (Figure 1A). *Gadd45a* was the top most significantly differentially expressed transcript in denervated gastrocnemius at 1 day postdenervation (mean log_2_ fold change [log_2_FC] = 2.94, FDR-corrected *P* = 3.21 × 10^–16^), and remained the most significantly differentially expressed transcript throughout the first 14 days postdenervation (mean log_2_FC, FDR-corrected *P* at 3 days: 4.98, 1.05 × 10^–23^; 7 days: 5.43, 9.81 × 10^–27^; 14 days: 5.67, 1.57 × 10^–27^). *Gadd45a* levels showed a modest but statistically significant decline at 90 days postdenervation (mean log_2_FC = 4.22, FDR-corrected *P* = 2.56 × 10^–23^). Time-series analysis revealed that *Gadd45a* was among the top 5 most differentially expressed genes detected in atrophying muscle across the entire 90-day interval postdenervation (Hotelling’s *T*^2^ statistic = 4903.43, distance = 5.99; Figure 1B). We validated this pattern of expression by quantitative PCR (qPCR) in gastrocnemius muscle from an independent C57BL/6J mouse tibial nerve denervation cohort (Figure 1C) and confirmed a corresponding increase in GADD45A expression at the protein level by Western analysis at 14 days postdenervation (Figure 1, D and E).

Using denervated human skeletal muscle samples obtained from patients with traumatic nerve injuries or acute flaccid myelitis, we confirmed that *GADD45A* expression was significantly increased in human denervated muscle compared with intact control muscle (Figure 1, F and G; mean fold change = 22.5; 95% CI 13.7, 37.1; *t* = 12.714, df = 31.629, *P* = 5.664 × 10^–14^), even when expression levels of *TRIM63* and *FBXO32*, the ubiquitin E3 ligases classically associated with the proteolytic degradation intrinsic to muscle atrophy, were low. This is consistent with observations in our tibial nerve denervation mouse model of neurogenic atrophy, wherein *Trim63* and *Fbxo32* showed significant induction during the first 7 or 14 days postdenervation, respectively, but then returned to baseline or below baseline levels (Figure 1A). The severity of nerve injury and loss of muscle function in the included cases were consistent with extensive denervation, which could explain the generally high level of *GADD45A* induction observed. These cases represent acute denervation injuries, with subsequent denervation durations ranging from 2 to 48 months, and evaluation of individual ΔCt values demonstrated that *GADD45A* expression remained sustained at remarkably high levels in a wide distribution of muscle groups even long after initial denervation compared with normal skeletal muscle (Figure 1H and Table 1).

### Gadd45a-null mice show significantly accelerated and more severe neurogenic atrophy.

**Figure 2:**
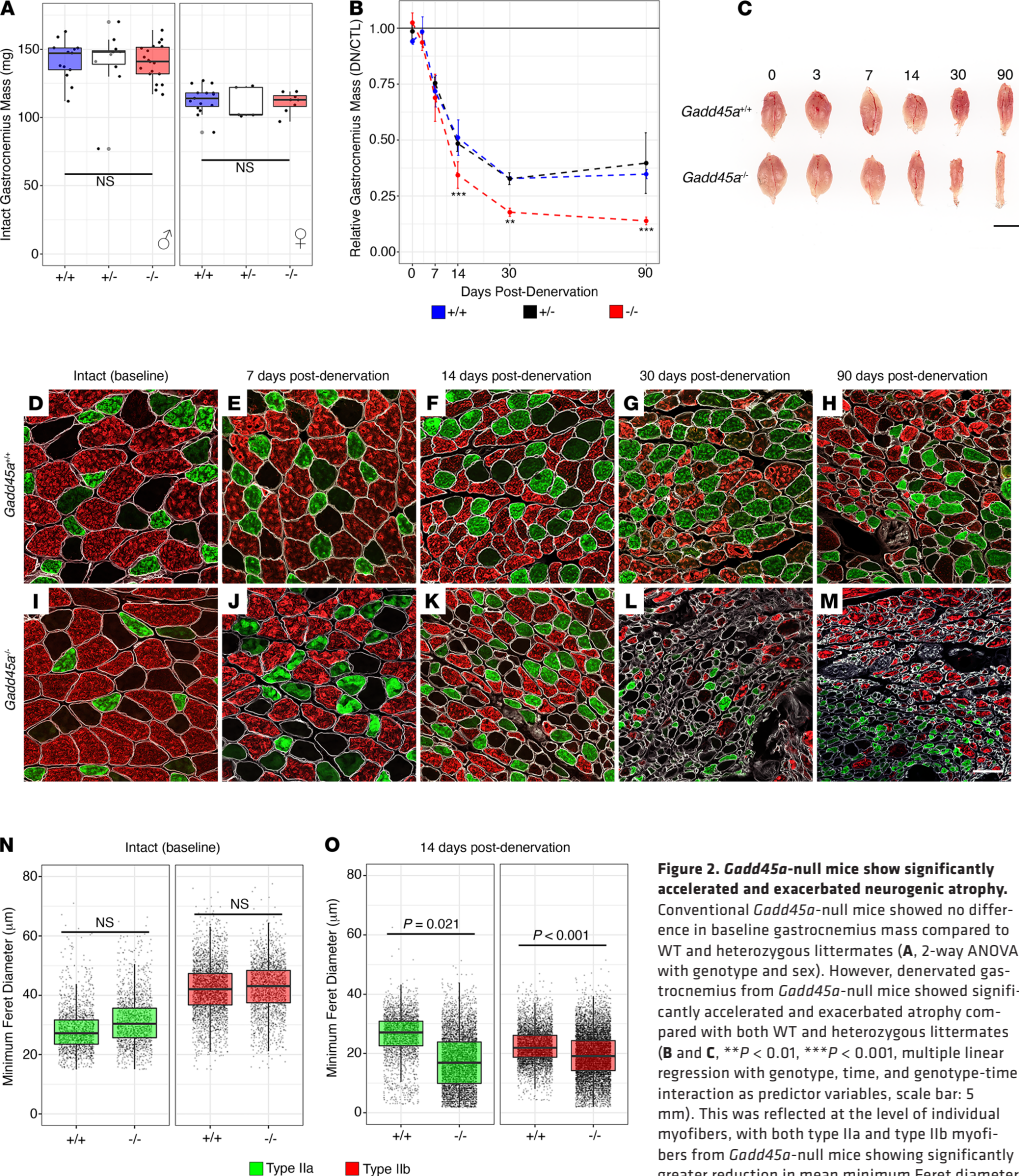
*Gadd45a*-null mice show significantly accelerated and exacerbated neurogenic atrophy. Conventional *Gadd45a*-null mice showed no difference in baseline gastrocnemius mass compared to WT and heterozygous littermates (**A**, 2-way ANOVA with genotype and sex). However, denervated gastrocnemius from *Gadd45a*-null mice showed significantly accelerated and exacerbated atrophy compared with both WT and heterozygous littermates (**B** and **C**, **P < 0.01, ***P < 0.001, multiple linear regression with genotype, time, and genotype-time interaction as predictor variables, scale bar: 5 mm). This was reflected at the level of individual myofibers, with both type IIa and type IIb myofibers from *Gadd45a*-null mice showing significantly greater reduction in mean minimum Feret diameter compared with WT littermates (**D**–**O**, 2-way ANOVA, scale bar: 100 μm). The box plots depict the minimum and maximum values (whiskers), the upper and lower quartiles, and the median. The length of the box represents the interquartile range.

Based on earlier studies suggesting that viral overexpression of GADD45A is sufficient to induce muscle atrophy, we obtained a conventional genetic knockout mouse model of *Gadd45a* (11) to evaluate whether preventing *Gadd45a* induction can ameliorate neurogenic atrophy. We did not detect any difference among genotypes (WT, heterozygous, and knockout) in baseline (intact, nondenervated) gastrocnemius mass (*n* = 16–28 for each genotype; 2-way ANOVA with genotype and sex as covariates, *P* < 0.001 for sex, *P* = 0.78 for genotype, *P* = 0.71 for genotype-sex interaction; Figure 2A). However, multiple linear regression with genotype, time, and genotype-time interaction as predictor variables indicated that, compared with WT littermates, *Gadd45a*-null muscle had a 25% ± 6% greater loss of mass by 14 days after denervation (*P* < 0.001), which was sustained to 29% ± 7% greater loss of mass at 90 days after denervation (*P* < 0.001) (mean ± SEM, *n* = 3–8 for each genotype at each time point; Figure 2, B and C). Heterozygous mice did not differ significantly from WT mice (WT vs. heterozygous gastrocnemius mass, genotype-time interaction: *P* = 0.90 at 7 days, *P* = 0.55 at 14 days, *P* = 0.96 at 72 days) (Figure 2B). Sex did not appear to significantly modify the relationship between mass and denervation duration or mass and genotype. The rate of mass loss differed significantly among knockouts versus WT mice during the first 14 days postdenervation (*P* < 0.001) but did not differ from 14–30 days (*P* = 0.50) or 30 to 72 days (*P* = 0.44) postdenervation (Figure 2B).

This accelerated atrophy in the absence of GADD45A was evident at the level of individual myofibers (Figure 2, D–M). Type IIa and type IIb myofibers differed in size at baseline in intact gastrocnemius (*P* = 0.002), but there was no difference in relative size of these 2 fiber types among WT versus *Gadd45a*-knockout littermates (Figure 2, D, I, and N; mean minimum Feret diameter, type IIa, 28.5 ± 7.4 μm for WT and 31.4 ± 8.1 μm for knockout; type IIb, 42.0 ± 8.5 μm for WT and 42.6 ± 8.6 μm for knockout; *P* = 0.28 for overall difference between genotypes, *P* = 0.42 for genotype-fiber type interaction, 2-way ANOVA with correction for intrareplicate correlation; *n* = 3 for each genotype). However, at 14 days postdenervation, both type IIa and IIb myofibers were significantly more atrophied in *Gadd45a*-null gastrocnemius muscle compared with WT muscle (Figure 2, F, K, and O, type IIa, 26.0 ± 7.5 μm for WT and 17.3 ± 9.0 μm for knockout, *P* = 0.02; type IIb, 22.5 ± 5.8 μm for WT and 19.3 ± 7.5 μm for knockout, *P* < 0.001, 2-way ANOVA with correction for intrareplicate correlation, *n* = 3 for each genotype).

Altogether, these findings suggest that *Gadd45a* induction after skeletal muscle denervation confers a protective effect against atrophy, as might be predicted for a stress-induced transcript.

### Denervation induces Gadd45a expression specifically in skeletal muscle myocytes.

We were unable to identify an antibody that specifically and reliably detects endogenous GADD45A in skeletal muscle cryosections. We therefore generated a V5 epitope–tagged *Gadd45a* allele also capable of conditional deletion, using CRISPR/Cas9 technology (Supplemental Figure 1A; supplemental material available online with this article; https://doi.org/10.1172/jci.insight.149381DS1). We confirmed the expected genomic modifications by Sanger sequencing (Supplemental Figure 1, B and C) and detected expression of V5-tagged protein at the expected molecular weight exclusively in denervated gastrocnemius muscle from C57BL/6J-*Gadd45a^em1Ahoke^*/Mmjax (*Gadd45a*^fl^) mice but not in intact (nondenervated) muscle or WT muscle (Supplemental Figure 1D).

**Figure 3:**
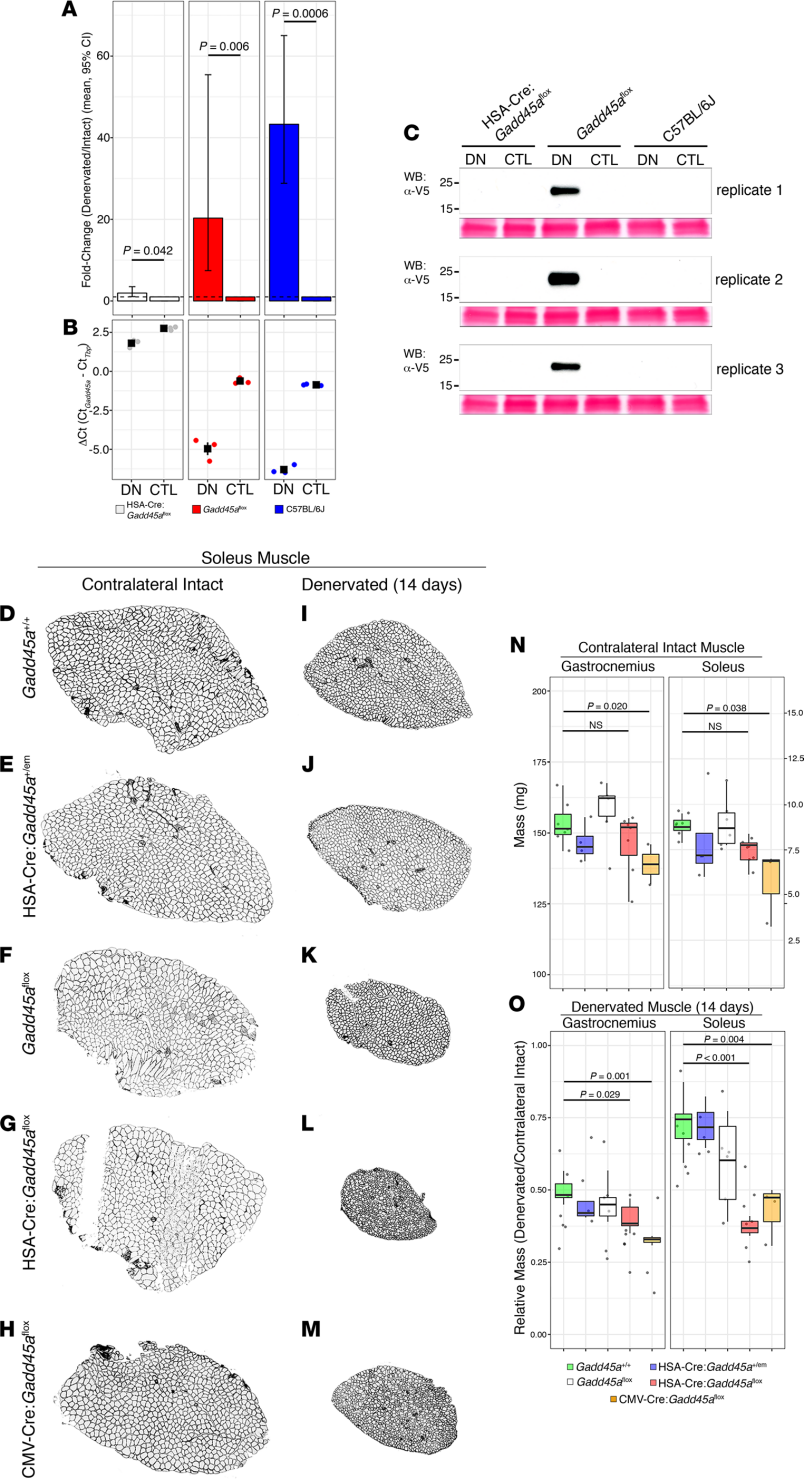
Denervation induces *Gadd45a* expression specifically in skeletal muscle myocytes. Skeletal muscle–specific deletion of *Gadd45a* (HSA-Cre:*Gadd45a*^fl^) essentially abolished its induction in denervated gastrocnemius compared with *Gadd45a*^fl^ and WT littermates (**A** and **B**, paired *t* test). Error bars in **A** show mean ± SEM. Accordingly, a target corresponding to endogenous V5 epitope–tagged GADD45A was detected in denervated gastrocnemius from *Gadd45a*^fl^ mice (but not contralateral intact muscle) but was abolished in denervated HSA-Cre:*Gadd45a*^fl^ gastrocnemius (**C**). Baseline gastrocnemius and soleus muscle mass from WT, heterozygous, *Gadd45a*^fl^, HSA-Cre:*Gadd45a*^fl^ (muscle-specific *Gadd45a* deletion), and CMV-Cre:*Gadd45a*^fl^ (ubiquitous *Gadd45a* deletion) did not significantly differ (**D**–**H** and **N**; soleus muscle shown, scale bar: 300 μm). However, at 14 days postdenervation, gastrocnemius and soleus muscle from HSA-Cre:*Gadd45a*^fl^ mice showed significantly greater loss of mass compared with WT, heterozygous, and *Gadd45a*^fl^ genotypes (**I**–**M** and **O**, 1-way ANOVA with Bonferroni’s correction). The degree of gastrocnemius and soleus mass loss in CMV-Cre:*Gadd45a*^fl^ mice does not significantly differ from HSA-Cre:*Gadd45a*^fl^ mice, consistent with the inference that the protective effect of *Gadd45a* induction is mediated entirely through its expression in skeletal muscle myocytes. The box plots depict the minimum and maximum values (whiskers), the upper and lower quartiles, and the median. The length of the box represents the interquartile range.

Denervation of mice carrying V5-tagged conditional *Gadd45a* revealed that the *Gadd45a* induction associated with denervation injury resulted exclusively from expression in skeletal muscle myocytes. We selectively excised the first 3 coding exons of *Gadd45a* from myonuclei by breeding *Gadd45a*^fl^ mice with a transgenic mouse line carrying Cre recombinase expression directed by the human skeletal actin (HSA) promoter: HSA-Cre79; B6.Cg-Tg(ACTA1-cre)79Jme/J. Mice with skeletal muscle–specific deletion of *Gadd45a* (HSA-Cre:*Gadd45a*^fl^) showed minimal induction of *Gadd45a* mRNA in denervated gastrocnemius compared with contralateral intact muscle at 14 days postdenervation (Figure 3, A and B) [*t*(2) = –4.7547, *P* = 0.0413, *n* = 3]. In contrast, *Gadd45a*^fl^ and WT mice showed significantly increased *Gadd45a* mRNA levels in denervated gastrocnemius at 14 days postdenervation (Figure 3, A and B) [*t*(2) = –12.8978, *P* = 0.006 and *t*(2) = –39.8712, *P* = 0.0006, respectively, *n* = 3 of each genotype]. The magnitude of *Gadd45a* induction appeared to differ between *Gadd45a*^fl^ and WT mice, although this did not achieve statistical significance [1-way ANOVA, *F*(2,6) = 95.54, *P* < 0.0001; *Gadd45a*^fl^ vs. WT *P* = 0.054]. The minimal degree of induction observed in HSA-Cre:*Gadd45a*^fl^ muscle differed significantly from both of the other genotypes [1-way ANOVA, *F*(2,6) = 95.54, *P* < 0.0001, HSA-Cre:*Gadd45a*^fl^ vs. *Gadd45a*^fl^ *P* < 0.001, HSA-Cre:*Gadd45a*^fl^ vs. WT *P* < 0.001].

These findings were confirmed at the protein level using an anti-V5 antibody to detect epitope-tagged GADD45A (Figure 3C). No target was detected in denervated or control muscle from C57BL/6J mice lacking the V5 epitope. However, an approximately 20 kDa protein corresponding to the expected molecular weight of V5 epitope–tagged GADD45A was detected in denervated gastrocnemius, but not contralateral intact gastrocnemius, from *Gadd45a*^fl^ mice. Importantly, this signal was completely abolished in mice with skeletal muscle–specific deletion of *Gadd45a* (HSA-Cre:*Gadd45a*^fl^). Altogether, these findings confirm that the GADD45A induction observed in denervated muscle occurs predominantly, and likely exclusively, in skeletal muscle myocytes.

### Gadd45a induction protects against neurogenic skeletal muscle atrophy.

Representative transverse sections from denervated and contralateral intact soleus muscle from *Gadd45a*^+/+^, HSA-Cre:*Gadd45a*^+/em^ (heterozygous deletion of Gadd45a from skeletal muscle), *Gadd45a*^fl^, HSA-Cre:*Gadd45a*^fl^, and CMV-Cre:*Gadd45a*^fl^ genotypes are shown in Figure 3, D–M. Baseline masses of the gastrocnemius and soleus muscles from age-matched and littermate WT, heterozygous, *Gadd45a*^fl^, and HSA-Cre:*Gadd45a*^fl^ mice did not differ among genotypes (Figure 3N) [1-way ANOVA, gastrocnemius: *F*(3,20) = 2.05, *P* = 0.1391; soleus: *F*(3,20) = 2.08, *P* = 0.1344; *n* = 4–7 per genotype]. At 14 days postdenervation, however, we observed a statistically significant difference in relative mass (denervated/contralateral intact) among genotypes for both gastrocnemius and soleus (Figure 3O) [1-way ANOVA, gastrocnemius: *F*(3,20) = 3.46, *P* = 0.0359; soleus: *F*(3,20) = 16.26, *P* = 1.37 × 10^–5^, *n* = 4–7 per genotype]. Post hoc analysis showed that the relative loss of gastrocnemius and soleus mass among WT, HSA-Cre:*Gadd45a*^em/+^, and *Gadd45a*^fl^ genotypes was similar (gastrocnemius: WT 0.50 ± 0.04, G*add45a*^fl^ 0.46 ± 0.06, HSA-Cre:*Gadd45a*^em/+^ 0.45 ± 0.07, *P* = 1.0 WT vs. HSA-Cre:*Gadd45a*^em/+^, *P* = 1.0 WT vs. *Gadd45a*^fl^; soleus: WT 0.73 ± 0.09, G*add45a*^fl^ 0.59 ± 0.16, HSA-Cre:*Gadd45a*^em/+^ 0.73 ± 0.08, *P* = 0.176 WT vs. *Gadd45a*^fl^, *P* = 1.0 WT vs. HSA-Cre:*Gadd45a*^em/+^). However, denervated gastrocnemius and soleus muscle from HSA-Cre:*Gadd45a*^fl^ mice showed significantly larger loss of mass compared with WT (gastrocnemius: 0.41 ± 0.04, *P* = 0.029; soleus: 0.38 ± 0.06, *P* < 0.001). These data further support the conclusion that GADD45A induction specifically in skeletal muscle myocytes confers a protective effect against neurogenic atrophy.

We further generated mice with ubiquitous deletion of *Gadd45a* using B6.C-Tg(CMV-cre)1Cgn/J mice. Approximately 50% of female CMV-Cre:*Gadd45a*^fl^ mice demonstrated dystocia with their first pregnancy, resulting in death, as previously described in a separate mouse model with ubiquitous targeted deletion of *Gadd45a* (11). The relative loss of gastrocnemius and soleus muscle mass among CMV-Cre:*Gadd45a*^fl^ mice did not differ significantly from HSA-Cre:*Gadd45a*^fl^ mice (Figure 3O).

### Absence of Gadd45a induction in skeletal muscle myocytes results in atrophy of multiple fiber types and apparent loss of fiber type identity.

**Figure 4:**
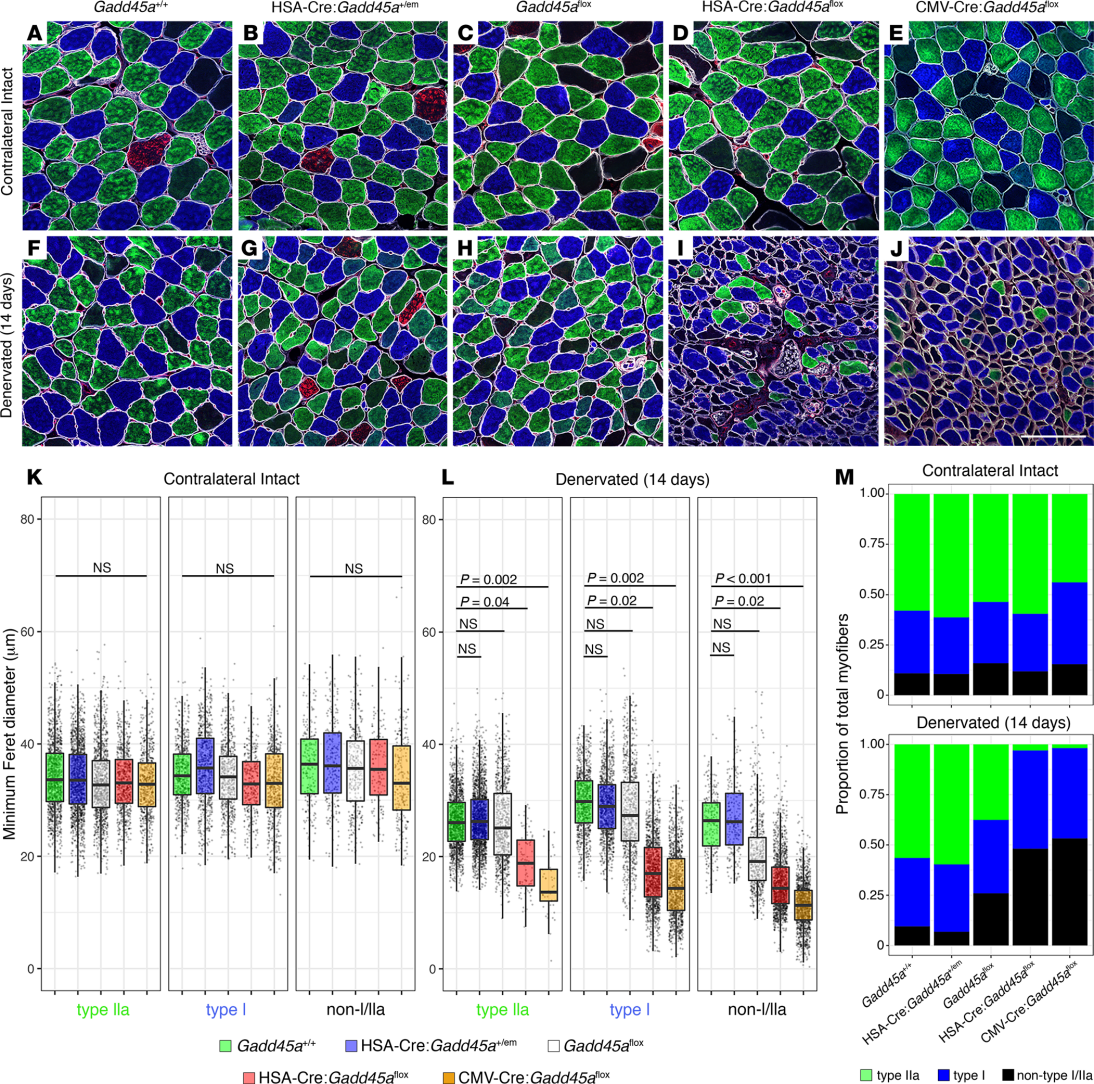
Absence of *Gadd45a* induction in skeletal muscle myocytes results in increased atrophy of multiple fiber types and loss of fiber type identity. Baseline minimum Feret diameters of type I, type IIa, and non–I/IIa myofibers did not differ among WT, heterozygous, *Gadd45a*^fl^, HSA-Cre:*Gadd45a*^fl^ (muscle-specific *Gadd45a* deletion), and CMV-Cre:*Gadd45a*^fl^ (ubiquitous *Gadd45a* deletion) genotypes (**A**–**E**, scale bar: 100 μm; **K**, multiple linear regression, with pairwise comparisons determined by linear combination). Type I, type IIa, and non–I/IIa myofibers from denervated soleus of HSA-Cre:*Gadd45a*^fl^ and CMV-Cre:*Gadd45a*^fl^ mice all showed significantly reduced minimum Feret diameters by 14 days postdenervation compared with all other genotypes (**F**–**J**, and **L**, multiple linear regression, with pairwise comparisons determined by linear combination). The box plots depict the minimum and maximum values (whiskers), the upper and lower quartiles, and the median. The length of the box represents the interquartile range. In addition, denervated soleus from HSA-Cre:*Gadd45a*^fl^ and CMV-Cre:*Gadd45a*^fl^ genotypes showed a significant increase (*P* < 0.001) in the proportion of non–I/IIa myofibers, with prominent loss of *Myh2*-expressing fibers (type IIa), suggesting accelerated loss of myofiber identity in the absence of GADD45A expression (**M**, Pearson’s χ^2^ test).

We performed myofiber morphometry analysis of intact soleus muscle and soleus muscle at 14 days after tibial nerve transection among congenic age-matched WT, heterozygous, *Gadd45a*^fl^, HSA-Cre:*Gadd45a*^fl^, and CMV-Cre:*Gadd45a*^fl^ mice, which enabled us to evaluate each transverse muscle section in its entirety. Minimum Feret diameters of type I, type IIa, and non–type I/IIa myofibers did not differ among any of the genotypes within intact, uninjured soleus muscle (Figure 4, A–E and K). At 14 days postdenervation, however, all 3 fiber types showed significantly reduced minimum Feret diameters in HSA-Cre:*Gadd45a*^fl^ and CMV-Cre:*Gadd45a*^fl^ soleus muscle compared with denervated WT, heterozygous, and *Gadd45a*^fl^ soleus (Figure 4, F–J and L) HSA-Cre:*Gadd45a*^fl^, type I: 18.9 ± 5.2 μm, *P* = 0.04, vs. WT; type IIa: 17.5 ± 6.2 μm, *P* = 0.02; non–type I/IIa: 15.3 ± 5.1 μm, *P* = 0.02; CMV-Cre:*Gadd45a*^fl^, type I: 14.7 ± 4.8 μm, *P* = 0.002; type IIa: 15.1 ± 6.1 μm, *P* = 0.002; non–type I/IIa: 11.4 ± 4.1 μm, *P* < 0.001; WT, type I: 26.4 ± 5.1 μm; type IIa: 29.8 ± 5.5 μm; non–type I/IIa: 26.0 ± 5.3 μm). Myofiber size for each respective myofiber type did not differ between denervated HSA-Cre:*Gadd45a*^fl^ and CMV-Cre:*Gadd45a*^fl^ soleus (*P* = 0.1, *P* = 0.36, and *P* = 0.12 for type I, type IIa, and non–type I/IIa, respectively), further suggesting that the entire effect of GADD45A induction is conferred through its activity in skeletal muscle myocytes. Type IIb myofibers are rare in the mouse soleus and were not quantified.

Although fiber type transition is known to occur in skeletal muscle because of chronic denervation and reinnervation, we unexpectedly observed that denervated soleus muscle from HSA-Cre:*Gadd45a*^fl^ and CMV-Cre:*Gadd45a*^fl^ mice showed a significant increase (*P* < 0.001) in the proportion of myofibers lacking expression of either myosin I or IIa compared with WT and heterozygous mice (48.1% and 53.3% compared with 9.6% and 6.9%, respectively; Figure 4M). These non–I/IIa fibers showed the most severe atrophy and were accompanied by significant loss of myofibers with type IIa identity in particular (3.0% and 1.8% type IIa myofibers remaining in denervated HSA-Cre:*Gadd45a*^fl^ and CMV-Cre:*Gadd45a*^fl^ soleus compared with 56.5% and 59.7% in denervated WT and heterozygous soleus, respectively). Consistent with observations of gastrocnemius/soleus mass and myofiber dimensions in the soleus, denervated *Gadd45a*^fl^ mice showed a proportion of non–I/IIa myofibers and relative loss of type IIa myofibers intermediate between that of WT/heterozygous mice and HSA-Cre:*Gadd45a*^fl^*/*CMV-Cre:*Gadd45a*^fl^ mice (26.0% non–I/IIa myofibers and 37.6% type IIa fibers). The relative proportions of each myofiber type were similar among all genotypes in intact, uninjured soleus.

## Discussion

A variety of stimuli are known to induce GADD45A expression, and the consequences of this induction appear to be cell type specific. Earlier studies detected increased expression of *Gadd45a* in denervated soleus, gastrocnemius, and/or triceps muscle from the SOD1^G86R^ and SOD1^G93A^ mouse models of ALS, as well as in soleus and extensor digitorum muscles from rodent models of upper or lower motor neuron transection (12). *Gadd45a* induction has also been observed in muscle from older individuals subjected to 5 days of bed rest (13). Increased *GADD45A* expression has been described in gene expression microarray analyses of deltoid (14, 15) and quadriceps (15) muscles from individuals with ALS. Higher levels of *GADD45A* expression observed at later stages of disease correlate with more severe and widespread muscle atrophy (14).

Congruent with these observations, we detected significantly increased *GADD45A* expression in human skeletal muscle samples from patients with traumatic nerve injury or acute flaccid myelitis with denervation durations ranging from 2 to 48 months (Figure 1, F–H), consistent with earlier suggestions that *GADD45A* induction in skeletal muscle is a direct response to denervation. Previous studies using virus-mediated overexpression of GADD45A in nondenervated muscle from C57BL/6J mice suggested that the expression of GADD45A alone was sufficient to trigger muscle atrophy (16–19). Our findings using a series of genetic models suggest *GADD45A* induction in denervated skeletal muscle delays the rate of atrophy and myofiber type transition, potentially even serving to preserve myofiber identity during chronic denervation.

*Gadd45a* induction has been observed in a variety of additional settings associated with muscle atrophy, including aging, muscle disuse, starvation, chronic obstructive pulmonary disease, and critical illness (20), and has been suggested to be required for muscle atrophy in starvation, immobilization, and denervation (18). In an immobilization model of muscle atrophy, this has been proposed to occur through GADD45A-mediated activation of MAP3K4 (MEKK4) via conformational release of an autoinhibitory domain from the MAP3K4 kinase domain, leading to phosphorylation and activation of downstream MAP kinase kinases and as-yet-unknown downstream effectors (17). These studies used electroporation of mouse tibialis anterior (TA) muscles with plasmids encoding siRNA targeting *Gadd45a* or GADD45A overexpression constructs, and the effects were studied at 1 week postimmobilization (17). Our studies using 3 genetic mouse models (ubiquitous targeted ablation, Cre-mediated skeletal muscle–specific deletion, and Cre-mediated ubiquitous deletion) in the otherwise normal physiological context of atrophying denervated muscle strongly support a model wherein *Gadd45a* induction is a protective modifier of myofiber atrophy and fate. This interpretation is further supported by the observation that *Gadd45a*^fl^ mice carrying a V5 epitope, which unintentionally appears to have yielded a modestly hypomorphic allele, showed an atrophy phenotype intermediate to that of *Gadd45a*-knockout and *Gadd45a*-heterozygous or WT mice. The V5 epitope comprises 14 amino acids while GADD45A itself comprises only 165 amino acids, and this apparent hypomorphic feature may relate to altered mRNA stability or altered interaction with effector proteins (e.g., the GADD45A N-terminus is known to interact with TET1, described below).

*Gadd45a* induction has also been observed in several models of neuronal injury, including ischemia, spinal cord transection, and sciatic nerve crush, and findings from all of these studies suggest that its upregulation has a protective role after neuronal insult (21–26). Robust *Gadd45a* induction was observed in rat dorsal root ganglion neurons (DRGs) within 1 day after spinal nerve ligation and persisted as long as the injured nerves were prevented from regenerating (27). shRNA-mediated knockdown of *Gadd45a* in DRGs via intrathecal infusion was associated with increased death of DRGs following spinal cord ligation, whereas transduction of cultured neonatal DRGs with herpes simplex virus expressing human GADD45A significantly enhanced survival (27). *Gadd45a* has been found to be upregulated in DRGs after sciatic nerve transection as well, where it has been suggested to participate in p53 signaling, apoptosis, cellular senescence, and/or MAP kinase signaling (28).

Beyond the neuromuscular system, GADD45A has also been shown to have a protective role in mouse models of ventilator-induced lung injury as well as radiation- and bleomycin-induced lung injury (29–31). *Gadd45a* expression is induced in endothelial cells in these models of lung injury, and *Gadd45a*-null mice (11) show reduced AKT signaling and more severe susceptibility to these forms of lung injury. In these mouse models, GADD45A deficiency was proposed to result in differential ubiquitination of AKT via reduced expression of the deubiquitinase UCHL1, resulting in both increased proteasomal degradation of AKT and decreased AKT phosphorylation/activation in response to mechanical stress (30, 31). Considering the known role of AKT signaling in mediating skeletal muscle hypertrophy, it will be of interest to evaluate whether the protective effect of GADD45A we observed in denervated muscle is also mediated at least in part through modulation of AKT signaling. *Gadd45a*-null mice also show more severe fibrosis in a mouse model of nonalcoholic steatohepatitis, a form of hepatic scarring that can lead to liver failure, suggesting a protective role in this condition as well (32). Finally, *Gadd45a* is downregulated in a variety of malignancies, including chronic myelocytic leukemia (33). Its induction in endothelial cells has been shown to suppress tumor angiogenesis via reduction of mTOR-mediated phosphorylation and activation of STAT3 and resulting VEGFa expression (34, 35).

How GADD45A confers a protective effect during neurogenic atrophy is presently unknown. GADD45A interacts with numerous diverse proteins and as a result is proposed to have many diverse functions. Recent studies suggest that p38α signaling positively regulates skeletal muscle atrophy, and mice with skeletal muscle–specific ablation of p38α or heterozygous inactivating mutations in p38 show reduced atrophy of TA and/or gastrocnemius muscle in sciatic nerve resection models of denervation atrophy (36, 37). GADD45A has been shown to bind directly to p38α, although this interaction appears to result in tissue-specific and opposing functions on p38 activity (38). A potentiating effect on p38 kinase activity has been observed in some studies, as suggested by impaired p38-mediated MK2 activation in cells lacking GADD45A (39) and a requirement for GADD45A in H-ras–mediated activation of p38 (40). GADD45A also indirectly regulates p38 signaling by binding and activating MEKK4 (41), leading to phosphorylation and activation of MKK3/MKK6, which leads to activation of p38 (42). However, GADD45A is not required for activation of p38 in all settings of cellular stress, as no reduction in JNK or p38 kinase activation was observed in *Gadd45a*-knockout mouse embryonic fibroblasts (MEFs) subjected to UV irradiation compared with WT MEFs (43). Furthermore, constitutive activation of T cell p38 has been observed in the absence of GADD45A, demonstrating that GADD45A can serve as a negative regulator of p38 activity as well (44).

Although the major degradative events of muscle atrophy have been classically associated with proteasomal degradation, in particular the E3 ubiquitin ligases TRIM63 and FBXO32, lysosomal degradation and autophagy also contribute. GADD45A is a negative regulator of autophagy, binding directly to BECN1 and thereby inhibiting its interaction with PIK3CK (45). GADD45A has also been shown to induce cell cycle arrest at the G2/M phase through interaction with cyclin B1 and induces cellular senescence in human fibroblasts (46–50). This could be pertinent as skeletal muscle myocytes are terminally differentiated cells, but experimentally forced cell cycle reentry (e.g., through overexpression of the skeletal muscle transcription factor MyoD, as occurs in denervated muscle) is associated with loss of skeletal muscle–specific gene expression (51).

Finally, GADD45A has a unique role in regulating methylation-dependent expression of specific target genes. This role emerged from an unbiased expression screen for DNA demethylases, and GADD45A overexpression was subsequently shown to promote demethylation and activation of methylation-silenced reporter genes. Premethylated CpG islands undergo active demethylation when transfected into embryonic stem (ES) cells; however, after *Gadd45a* knockdown in ES cells, these sequences retained substantial degrees of methylation (52). Deletion of 3 GADD45 family members (*Gadd45a*, *Gadd45b*, and *Gadd45c*), all of which function in demethylation, in ES cells is associated with locus-specific hypermethylation, primarily found in intronic and intergenic regions (53). Deletion of *Gadd45a/b* in mouse ES cells leads to hypermethylation at sites mostly overlapping with sites known to be dependent on thymine DNA glycosylase (TDG) for demethylation. GADD45A is now known to interact with key enzymes involved in DNA demethylation, including TET methylcytosine dioxygenase 1 (TET1) and TDG (54–57). Active demethylation involves TET1-mediated 5mC oxidations followed by base-excision or nucleotide-excision repair conversions of oxidized intermediates to cytosines (58, 59). GADD45A is thought to promote TET1 activity and/or recruit key components of DNA repair at specific genomic loci, leading to cytosine demethylation within targeted CpG islands and activation of gene expression (56, 57, 60–62). GADD45A has also been described to bind directly to R-loops at CpG islands, leading to recruitment of TET1 and associated demethylation machinery to mediate local DNA demethylation (63). *Gadd45a* deficiency has been shown to be associated with hypermethylation of only a small number of previously mapped 5hmC- and 5fC-enriched regions in ES cells, suggesting that GADD45A may contribute to the regulation of only a small subset of TET-TDG regulated genomic targets (57). Altogether, these observations highlight the possibility that the effects of GADD45A deficiency on skeletal muscle atrophy observed in our murine genetic series may result from epigenetically dependent effectors.

Fiber type transition is well-known to occur in denervated muscle, particularly in conditions where chronic denervation and reinnervation processes are present, and this transition typically reflects fiber type identity driven by the activity pattern of the reinnervating axon. We observed that *Gadd45a* deficiency also results in loss of myofiber identity during denervation. Whether this reflects enhanced degradation of sarcomeric components related to atrophy or authentically failed preservation of myofiber identity warrants further exploration and could have implications for the processes that seemingly permanently alter myofiber phenotype during chronic denervation and that lead to impaired reinnervation capacity and functional restoration.

In summary, our work shows that a naturally occurring negative feedback mechanism exists in denervated skeletal muscle with the potential to modify the severity of atrophy and fate of individual denervated myofibers. Ongoing studies are underway to define the upstream and downstream signaling context within which GADD45A confers protection, with the intention of identifying targets that may be further harnessed to protect muscle from atrophy. This work could offer insight into pathways that may be modulated to successfully limit skeletal muscle atrophy, potentially preserving the capacity for muscle function and lengthening the window during which functional reinnervation may be achieved.

## Methods

### Animal husbandry.

*Gadd45a*-null mice were previously described (11). Genotyping was performed with genomic DNA extracted from tail biopsies using the REDExtract-N-Amp tissue lysis and PCR kit (MilliporeSigma, catalog XNAT) using the following primers: *Gadd45a*-mutant: 5′-AGAACGAGATCAGCAGCCTCT-3′; *Gadd45a*-same: 5′-GAAGACCTAGACAGCACGGTT-3′; *Gadd45a*-WT: 5′CCTCTGCTTACCTCTGCACAA-3′. Thermocycling conditions were 94°C 3 minutes, 35 cycles of 94°C 30 seconds, 54°C 30 seconds, 72°C 1 minute, and final extension at 72°C for 10 minutes. Expected product sizes were 324 bp (WT) and 211 bp (knockout). B6.Cg-Tg(ACTA1-cre)79Jme/J mice (HSA-Cre79, JAX 006149) and B6.C-Tg(CMV-cre)1Cgn/J mice (CMV-Cre, JAX 006054) were obtained from The Jackson Laboratory. Cre genotyping was performed using the following primers: *Cre*-F: 5′-ATTGCTGTCACTTGGTCGTGGC-3′; *Cre*-R: 5′-GGAAAATGCTTCTGTCCGTTTGC-3′; *Actin*-F: 5′-GATGACGATATCGCTGCGCTGGTCG-3′; *Actin*-R: 5′-GCCTGTGGTACGACCAGAGGCATACAG-3′. Thermocycling conditions were 94°C 2 minutes, 30 cycles of 94°C 30 seconds, 60°C 30 seconds, 72°C 1 minute, and final extension at 72°C for 10 minutes. Expected product sizes were 207 bp (cre) and 1000 bp (actin). All oligonucleotides were synthesized by Integrated DNA Technologies (IDT). Animal subjects were housed in a controlled environment with a 12-hour light/12-hour dark cycle with ad libitum access to water and food (Envigo 2018 SX).

### CRISPR/Cas9-mediated generation of conditional/epitope-tagged Gadd45a mice.

Guide RNAs (gRNAs) targeting intronic regions flanking the first 3 coding exons of murine *Gadd45a* (NCBI Reference Sequence NC_000072.6:c67039407-67033096, *Mus musculus* strain C57BL/6J) were designed using the web-based CRISPR design tool of MIT (https://crispr.mit.edu). Two of the top gRNAs (score ≥ 90) were selected for use as follows: *Gadd45a*-gRNA-5′: 5′-CAGCACCACCTTCGTCC^CGT-3′; *Gadd45a*-gRNA-3′: 5′-CGGAGCGTGTCTAAGCT^CGT-3′. Alt-R CRISPR/Cas9 crRNA oligonucleotides and a 1.5 kb single-stranded donor DNA megamer containing the desired 5′ *loxP*, 3′ *loxP*, a V5 epitope tag introduced directly after the start codon, and 180 nucleotide flanking regions were synthesized by IDT. TheV5 epitope sequence was as follows: 5′-GGTAAGCCTATCCCTAACCCTCTCCTCGGTCTCGATTCTACG-3′. Pronuclear injection of 1-cell C57BL/6J embryos (The Jackson Laboratory) was performed by the Johns Hopkins University Transgenic Core using standard microinjection techniques (64) using a mix of Cas9 protein (30 ng/μL, PNABio), tracrRNA (0.6 μM, Dharmacon), crRNA (0.6 μM, IDT), and ssDNA oligo (5 ng/μL, IDT) diluted in RNase-free injection buffer (10 mM Tris-HCl, pH 7.4, 0.25 mM EDTA). Injected embryos were transferred into the oviducts of pseudopregnant ICR females (Envigo) using the technique described in Nagy et al. (64). Founders were screened for 5′ *loxP* and V5 epitope insertion using the following primers: *Gadd45a*-*loxP*-F3: 5′-AGGACACTTGAACCACTGCAA-3′ and *Gadd45a*-V5-R: 5′-CAGGCACACTTACCTTTCGGT-3′; expected product sizes were 510 bp (unmodified) and 586 bp (modified). The 3′ *loxP* insertion was screened using the following primers: *Gadd45a*-*loxP*-F1: 5′-TGCTGCTACTGGAGAACGAC-3′ and *Gadd45a*-*loxP*-R2: 5′-AATTAGCCACGCGAGGTTGT-3′; expected product sizes were 285 bp (unmodified) and 319 bp (modified). PCR fragments from founding lines were purified using AMPure XP beads (Beckman Coulter) and verified by Sanger sequencing. A homozygous founder carrying both modified sites was backcrossed 3 generations to C57BL/6J mice. This line has been deposited with the Mutant Mouse Resource and Research Center at The Jackson Laboratory and is available as stock 050634-JAX (C57BL/6J-*Gadd45a^em1Ahoke^*/Mmjax, abbreviated as *Gadd45a*^fl^). Genotyping was performed using the REDExtract-N-Amp tissue lysis and PCR kit (MilliporeSigma, catalog XNAT) and the following thermal cycling conditions: 94°C 3 minutes; 35 cycles of 94°C 30 seconds, 54°C 30 seconds, 72°C 1 minute; and final extension at 72°C for 10 minutes.

### RNA-Seq library preparation, sequencing, and bioinformatics analysis.

The murine denervation data set was described previously (10). RNA-Seq was carried out using TrueSeq RiboZero gold (stranded) kit (Illumina, catalog 20020597). Libraries were indexed and sequenced over 18 lanes using HiSeq4000 (Illumina) with 69 bp paired-end reads. Quality control was performed on base qualities and nucleotide composition of sequences using FastQC version 0.11.5. Paired-end reads were aligned to the most recent *Mus musculus* mm10 reference genome (GRCm38.75) using the STAR spliced read aligner (version 2.4.0; ref. 65). Total counts of read-fragments aligned to known gene regions within the mouse (mm10) refSeq (refFlat version 07.24.14) reference annotation were used as the basis for quantification of gene expression. Fragment counts were derived using HTSeq (version 0.6.0) and the mm10 refSeq transcript model (66). Low-count transcripts were filtered, and count data were normalized using the method of trimmed mean of M values (67) followed by removing unwanted variation using Bioconductor package RUVseq (68) with k value of 1. Differentially expressed genes (FDR < 0.1) were then identified using the Bioconductor package limma with voom function to estimate mean-variance relationship, followed by empirical Bayes moderation (69–71). Pairwise comparisons between denervated and contralateral intact muscle at each time point were used as the basis for model contrasts (NCBI’s Sequence Read Archive, https://www.ncbi.nlm.nih.gov/sra/?term=SRP196460).

### Human skeletal muscle specimens.

Denervated human skeletal muscles were obtained from specimens discarded during elective nerve repair surgeries at Johns Hopkins Hospital. Histologically normal control skeletal muscle specimens were obtained from archived samples available from the Johns Hopkins Neuromuscular Pathology Lab and were age and site matched to denervated samples to the extent possible. Specimens were frozen in liquid nitrogen–cooled isopentane and stored at –80°C until processed for RNA isolation.

### Tibial nerve denervation surgery.

Mice were anesthetized with 1.5% isoflurane/2% oxygen using a VetEquip inhalation system. The left hind limb was shaved and sterilized, and a 1 cm incision was introduced in the skin overlying the dorsal thigh. Myofascial planes were gently separated to reveal the sciatic nerve. The tibial nerve branch was identified at its distal branch point and gently separated from the sciatic and peroneal nerves, then ligated proximally and distally using a 10 to 0 polyamide monofilament suture. The tibial nerve was then transected, the nerve length between ligatures carefully resected, and the proximal stump sutured to the biceps femoris muscle to prevent distal reinnervation. The incision was then closed using stainless steel wound clips. Mice were monitored for recovery from anesthesia and then returned to their home cages.

### RNA isolation.

Skeletal muscle was homogenized in TRIzol (Ambion, catalog 15596018) using RNase-free stainless steel beads (Next Advance, catalog SSB02-RNA). Homogenates were centrifuged at 7840*g* for 10 minutes to pellet debris, and RNA was purified from the TRIzol supernatant using a Direct-Zol RNA mini purification kit with on-column DNase digestion (Zymo Research, catalog R2072). RNA integrity number (RIN) was assayed using an Agilent 2100 Bioanalyzer. RIN for RNA isolated from fresh frozen human skeletal muscle was 7.3 ± 0.8 (normal muscle, mean ± SD) and 7.7 ± 1.1 (denervated muscle, mean ± SD), with no significant difference in RIN between denervated and control samples by Welch’s *t* test, *t*(24.60) = –1.25 (*P* = 0.22). RIN for RNA isolated from mouse skeletal muscle was not regularly assayed but was found to routinely be approximately 8.0 in previous experiments using identical methods, with no significant difference in RIN by denervation status (10).

### qPCR.

A total of 0.45 to 1 μg RNA was used for cDNA synthesis using the high-capacity cDNA reverse-transcription kit containing random primers (Thermo Fisher Scientific catalog 4368814), with the following cycling conditions: 25°C for 10 minutes, 37°C for 120 minutes, 85°C for 5 minutes. qPCR was performed using TaqMan gene expression assays (Thermo Fisher Scientific catalog 4331182) with TaqMan Fast gene expression master mix (Thermo Fisher Scientific catalog 4444554) and 10–50 ng cDNA equivalents of template DNA. The following FAM-MGB-labeled TaqMan primer/probe sets were used: *Gadd45a*, Mm00432802_m1; *Trim63*, Mm01185221_m1; *Fbxo32*, Mm00499523_m1; *GADD45A*, Hs00169255_m1; *TRIM63*, Hs00822397; *FBXO32*, Hs01041408_m1. Real-time PCR was performed using a StepOnePlus real-time PCR system (Applied Biosystems, Thermo Fisher Scientific). We determined relative quantities using the comparative C_T_ (ΔΔC_T_) method (72, 73), using VIC-MGB–labeled *TBP* (human, catalog 4448489, Hs00427620_m1) or VIC-MGB–labeled *Tbp* (mouse, catalog 4448489, Mm01277042_m1) as endogenous controls for normalization (74). All reactions were performed in technical triplicates with the number of biological replicates as indicated in the text/figures.

### Immunofluorescence and myofiber morphometry.

Gastrocnemii were frozen in OCT in liquid nitrogen–cooled isopentane (75), then sectioned at 10 μm. Midbelly transverse sections were blocked with Mouse-on-Mouse blocking reagent in PBS (1:40 dilution, Vector Laboratories, catalog MKB-2213) at room temperature for 1 hour, then incubated overnight at 4°C with a mixture of BA-D5 supernatant (1:100, myosin heavy chain type I), SC-71 supernatant (1:100, myosin heavy chain type IIa), BF-F3 concentrate (1:100, myosin heavy chain type IIb; all from the Developmental Studies Hybridoma Bank), and rat anti-laminin (1:1000, MilliporeSigma, catalog L0663) in 1% BSA/PBS. Sections were then washed 3 times 5 minutes in PBS and incubated with a mixture of the following secondary antibodies (all at 1:500) for 2 hours at room temperature: goat anti-mouse IgG2b–DyLight-405, IgG1–Alexa Fluor 488, IgM–Alexa Fluor 594 (all from Jackson ImmunoResearch, catalog numbers 115-475-207, 115-545-205, and 115-585-075, respectively), and goat anti–rat-IgG–Alexa Fluor 647 (Thermo Fisher Scientific, catalog A-21247), diluted in 1% BSA/PBS. Sections were washed 3 times 5 minutes in PBS and coverslipped using ProLong Gold antifade (Thermo Fisher Scientific, catalog P36930). Images were collected using an Airyscan 800 confocal microscope (Zeiss). For myofiber morphometry, transverse gastrocnemius and soleus sections were imaged in their entirety using a BZ-X700 fluorescence microscope (Keyence). Myofiber minimum Feret diameters were determined using QuantiMus (76). For QuantiMus analysis, more than 2000 myofibers of each fiber type were measured from each of 3 to 4 biological replicates for each genotype, with the exception of denervated HSA-Cre:*Gadd45a*^fl^ and CMV-Cre:*Gadd45a*^fl^ soleus muscle, which showed a significant reduction in the population of type I myofibers.

### Western analysis.

Skeletal muscle was homogenized using steel beads (Next Advance, catalog SSB02-RNA) in ice-cold T-PER buffer (Thermo Fisher Scientific, catalog 78510) containing protease inhibitors (Roche, catalog 11697498001). Lysates were centrifuged at 7840*g* for 20 minuters at 4°C, and protein concentration was measured by BCA assay (Thermo Fisher Scientific, catalog 23227). A total of 50 μg protein lysates denatured in SDS buffer were boiled for 5 minutes, electrophoresed through 4% to 20% polyacrylamide gels in tris-glycine SDS running buffer (Thermo Fisher Scientific, catalog LC2675), and then transferred to nitrocellulose membranes in transfer buffer containing 20% methanol for 30 minutes at 100 V. Protein loading and transfer were evaluated by Ponceau S staining (Cell Signaling Technology, catalog 59803; ref. 77), and membranes were then blocked with 5% nonfat milk in TBS-Tween (TBST) for 1 hour at room temperature. Membranes were incubated overnight at 4°C in 5% nonfat milk with TBST containing goat anti-V5 antibody (1:1000, Abcam, catalog ab9137, lot GR172741) or rabbit anti-GADD45A (1:1000, Abcam, catalog ab180768, lot GR3218627). Membranes were washed in TBST and incubated for 1 hour at room temperature with 5% nonfat milk with TBST containing HRP-conjugated rabbit anti-goat secondary antibody (1:2500, Thermo Fisher Scientific, catalog R-21459) or goat anti-rabbit secondary antibody (1:3000, Amersham, catalog 934). Membranes were then incubated with ECL (GE, now Cytiva, catalog GERPN3243) or Femto (Thermo Fisher Scientific, catalog 34095) reagents, visualized using Hyperfilm ECL (Amersham, catalog GE28-9068-38), and scanned.

### Statistics.

Two-group comparisons were made using 2-sided Welch’s *t* test. One-way ANOVA with post hoc Bonferroni’s correction was used for comparisons having more than 2 groups. Skeletal muscle masses were compared using a linear regression model with genotype, sex, and time as covariates and an interaction term between genotype and time. Skeletal muscle myofiber minimum Feret diameters were compared using a linear regression model with genotype and myofiber type as covariates and an interaction term between genotype and myofiber type, with correction for intra-animal correlation. Myofiber proportions were compared using Pearson’s χ^2^ test. Statistical analyses were performed using R version 3.5.1 (78) and/or Stata version 11.2 (79). Data are expressed as means ± SD, means ± SEM, or means with 95% CI as indicated. *P* < 0.05 was considered statistically significant.

### Study approval.

Human skeletal muscle specimen collection was approved by the Johns Hopkins School of Medicine Institutional Review Board (IRB 00081649), and all samples were obtained with written informed consent from the patient or guardian. All mouse experiments were carried out under protocols approved by the Johns Hopkins University Animal Care and Use Committee (MO17M98, MO20M28).

## Author contributions

JTE and AH designed the study; JTE, DK, and AEJ conducted experiments; JTE, RK, DN, AEJ, GC, and AH analyzed data; JTE wrote the manuscript; and JTE and AH edited the manuscript.

## Supplementary Material

Supplemental data

## Figures and Tables

**Table 1:**
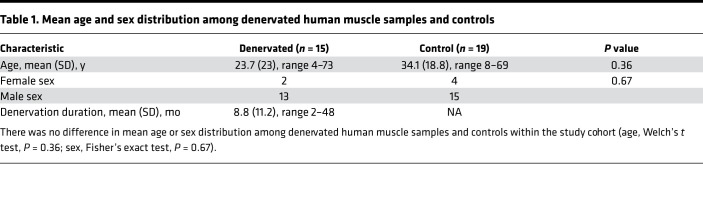
Mean age and sex distribution among denervated human muscle samples and controls

## Version 1 06/15/2021

In-Press Preview

## Version 2 07/08/2021

Electronic publication
