# Supplementary material for: GADD45A is a protective modifier of neurogenic skeletal muscle atrophy
Source: JCI Insight. 2021 Jul 8;6(13):e149381. doi: 10.1172/jci.insight.149381 (PMC8410074; doi:10.1172/jci.insight.149381)
Supplement: Supplemental data [file jciinsight-6-149381-s166.pdf]

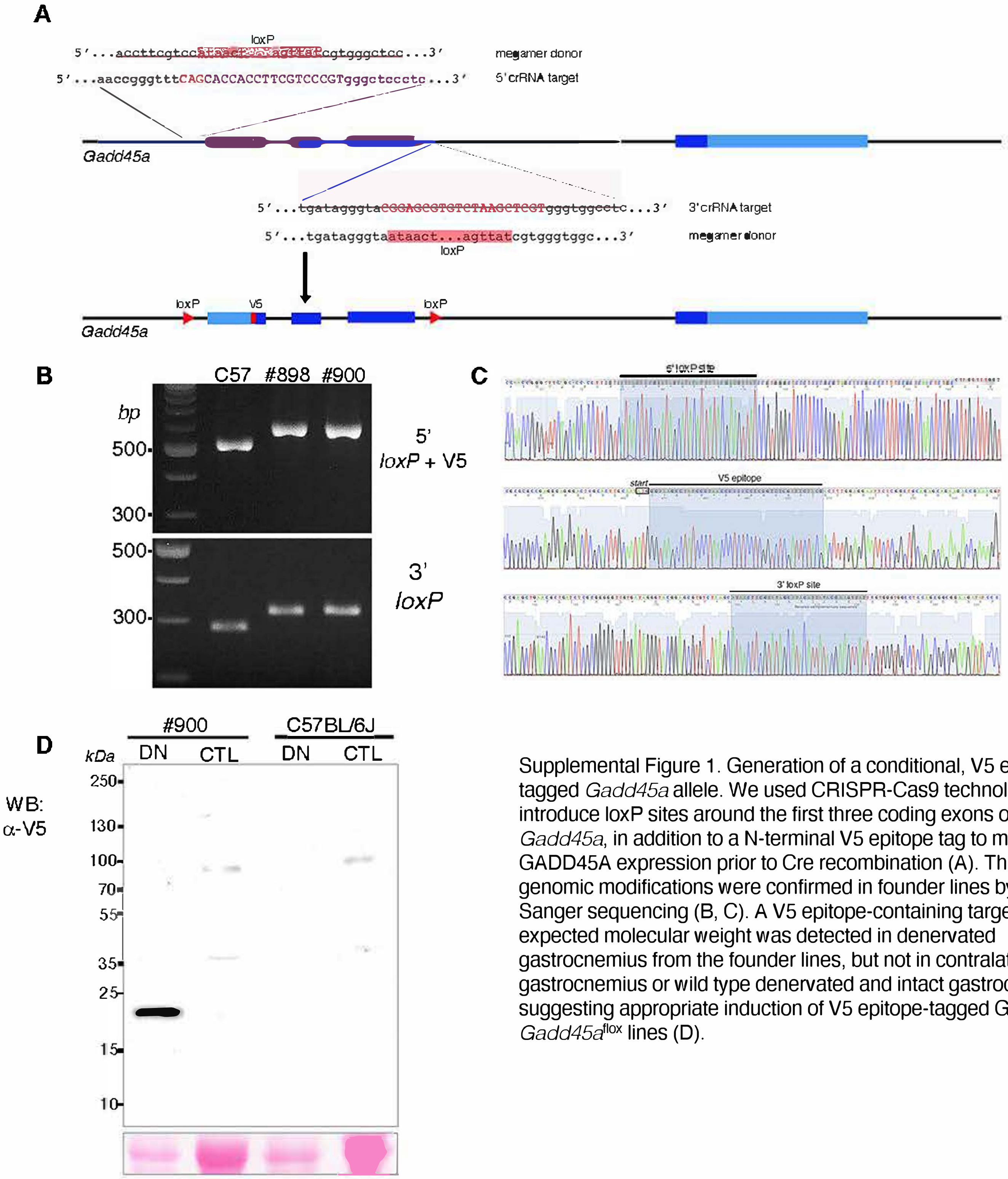

Supplemental Figure 1. Generation of a conditional, V5 epitope-tagged *Gadd45a* allele. We used CRISPR-Cas9 technology to introduce loxP sites around the first three coding exons of murine *Gadd45a*, in addition to a N-terminal V5 epitope tag to monitor GADD45A expression prior to Cre recombination (A). The expected genomic modifications were confirmed in founder lines by PCR and Sanger sequencing (B, C). A V5 epitope-containing target of the expected molecular weight was detected in denervated gastrocnemius from the founder lines, but not in contralateral intact gastrocnemius or wild type denervated and intact gastrocnemius, suggesting appropriate induction of V5 epitope-tagged GADD45A in *Gadd45a*^{flox} lines (D).
